# Monitoring Age-Related Changes in Gait Complexity in the Wild with a Smartphone Accelerometer System

**DOI:** 10.3390/s24227175

**Published:** 2024-11-08

**Authors:** Vincenzo E. Di Bacco, William H. Gage

**Affiliations:** School of Kinesiology and Health Science, York University, Toronto, ON M3J 1P3, Canada; whgage@yorku.ca

**Keywords:** nonlinear dynamics, statistical persistence, entropy, variability, wearables, free-living walking, adaptability

## Abstract

Stride-to-stride fluctuations during walking reflect age-related changes in gait adaptability and are estimated with nonlinear measures that confine data collection to controlled settings. Smartphones, with their embedded accelerometers, may provide accessible gait analysis throughout the day. This study investigated age-related differences in linear and nonlinear gait measures estimated from a smartphone accelerometer (SPAcc) in an unconstrained, free-living environment. Thirteen young adults (YA) and 11 older adults (OA) walked within a shopping mall with a SPAcc placed in their front right pants pocket. The inter-stride interval, calculated as the time difference between ipsilateral heel contacts, was used for dependent measures calculations. One-way repeated-measures analysis of variance revealed significant (*p* < 0.05) age-related differences (mean: YA, OA) for stride-time standard deviation (0.04 s, 0.05 s) and coefficient of variation (3.47%, 4.16%), sample entropy (SaEn) scale 1 (1.70, 1.86) and scale 3 (2.12, 1.80), and statistical persistence decay (31 strides, 23 strides). The fractal scaling index was not different between groups (0.93, 0.95), but exceeded those typically found in controlled settings, suggesting an upregulation in adaptive behaviour likely to accommodate the increased challenge of free-living walking. These findings support the SPAcc as a viable telehealth instrument for remote monitoring of gait dynamics, with implications for unsupervised fall-risk assessment.

## 1. Introduction

Reduced gait complexity is a signature of age-related changes in neuromuscular function and an increased risk of falls among older adults [1,2]. The Loss of Complexity hypothesis [3] posits that reduced complexity is due to the breakdown in the components or connections between components comprising the gait control system, diminishing the walker’s adaptability. Increased gait variability, estimated with linear measures, is generally considered a decline in gait performance and a predictor of falls in older adults [4,5,6]. However, nonlinear approaches to gait analysis appear to be more sensitive in detecting diminished adaptive capacity between healthy and unhealthy states [1]. The Optimal Movement Variability (OMV) model [7] suggests that healthy states reveal variability that is situated between the extremes of order and disorder or ‘controlled disorder’, and comprises a mixture of many available functional movement patterns that the individual cycles through. In terms of gait, the OMV model suggests that the walker generates many appropriate stepping strategies, and when needed, can transition to a strategy based on predicted or current imposed constraints. Therefore, disruptions in gait dynamics can manifest as both overly disorganized and overly organized patterns, indicating poor adaptability.

The fractal scaling index (FSI), calculated using detrended fluctuation analysis (DFA), quantifies long-range correlations in the inter-stride interval (ISI) series [8]. Healthy young adults typically demonstrate a stride-time FSI value between 0.75 and 0.85, indicating statistical persistence, while older adults exhibit FSI values closer to 0.5, indicating a disorganized pattern associated with fall risk [8,9,10]. Statistical persistence is a hallmark of adaptive systems, with an FSI value of 1.0 suggesting optimal adaptability [11,12]. For example, walking speeds greater or less than self-selected, as well as asymmetric walking, increase FSI values [11,13], which is likely to accommodate the imposed challenge.

Approximate entropy (ApEn) and sample entropy (SaEn) assess statistical regularity, with greater values suggesting a larger repertoire of stepping strategies available for the walker, thereby reflecting greater adaptability [12,14,15]. However, ApEn and SaEn only operate on a single scale, prompting the development of multiscale entropy (MSE) and the complexity index (CI) [16]. Recently, new measures termed “statistical persistence decay” (SPD) and “entropic half-life” (EnHL) have been introduced to quantify the stride number at which the stride-time series loses statistical persistence and regularity, respectively [17,18]. However, such measures have only been tested among healthy young adults in controlled environments [17,18].

The estimation of gait complexity requires hundreds of consecutive strides, thereby restricting data collection to controlled walking settings [19]. However, such settings may not represent stride patterns, as one freely ambulates throughout the day and ‘in the wild’. For example, the stride-time FSI among those living with Parkinson’s disease during treadmill walking was found to be similar to that of healthy young adults, while young adults demonstrated a decrease in statistical persistence during treadmill walking compared with overground walking [20]. Our previous work demonstrated similar findings among young adults, showing that compared with overground walking, treadmill walking revealed a decrease in stride-time statistical persistence and statistical regularity, most likely due to the confines of the treadmill belt dimensions and fixed-pace speed [19].

Although wearable inertial measurement unit sensors are suitable for recording the extended walking bouts (>200 strides) necessary for the application of nonlinear methods during free-living walking, few studies have done so, despite the potential these methods offer for estimating adaptability [21,22]. Moreover, wearable sensors must be provided to participants and require secure attachment to the user, which may not be feasible for long-term monitoring. Smartphones, with their on-board accelerometers, have accurately and reliably estimated both linear (e.g., stride time, gait speed) and nonlinear (e.g., FSI, SaEn) gait measures in controlled settings [23,24,25,26,27]. They have also shown promise in free-living environments for estimating linear measures [28,29,30]. However, to our knowledge, researchers have yet to explore nonlinear gait dynamics in these environments estimated with a smartphone. The current study implemented a smartphone accelerometer (SPAcc) system to estimate age-related changes in gait variability during free-living walking. We hypothesized that older adults would demonstrate greater linear variability, reduced gait complexity and statistical regularity, as well as earlier losses in statistical persistence and regularity, compared with young adults.

## 2. Materials and Methods

### 2.1. Participants

Thirteen healthy young adults (YA) (7F/6M; mean ± standard deviation (SD); age: 28.3 ± 3.7 years of age; weight: 72.6 ± 17.3 kg; height: 1.74 ± 0.1 m) and 11 healthy older adults (OA) (7F/4M; mean ± SD; age: 68.7 ± 2.8 years of age; weight: 76.9 ± 8.7 kg; height: 1.67 ± 0.11 m) volunteered to participate. Sample size was calculated a priori based on the FSI mean and variance values of YA and OA groups reported in Hausdorff et al. [1], with 80% power and an alpha of 0.05, resulting in n = 11 for each group. The FSI was used to calculate the sample size, as that was our main complexity measure. Each participant provided written informed consent prior to participation. The university research ethics board granted approval for the study (certificate# STU 2019-091). To assess study eligibility and physical activity level, all participants completed a screening questionnaire, the Activities-specific Balance Confidence (ABC) Scale questionnaire, and a fear of falling question (Yes; No; Somewhat). Inclusion criteria included the following: adults between 18 and 35 years of age, or ≥65 years of age; the ability to perform repeated 10 min walking bouts; no fear, or somewhat fearful of falling; an ABC score ≥ 67% [31]; a self-reported fall history of <2 falls in the previous 12 months [22]; and no neurological or musculoskeletal conditions or injuries within the previous six months that might affect gait performance. Participants were considered physically active if they performed ≥150 min of weekly moderate-to-vigorous physical activity [32,33]. Participant characteristics are described in Table 1.

### 2.2. Protocol

Participants visited a local shopping mall and wore comfortable walking shoes and full-length pants with front pockets. A Google smartphone (Pixel 2, Mountain View, CA, USA), with a custom-built application to access the embedded tri-axial accelerometer, was used as the SPAcc for all participants while sampling at 100 Hz. Participants were asked to complete four laps at their comfortable walking speed along the outermost corridors forming a large loop within the shopping mall within a two-hour period; the SPAcc was placed pointing downwards in their front right pant pocket. Each lap was approximately 12 min in duration. Participants were instructed to behave as they normally would during a typical mall visit (e.g., browse, shop, eat) while completing the four laps.

### 2.3. Data Processing

All data were processed using Matlab (R2021b, Mathworks Inc., Natick, MA, USA). Based on previous work, only the vertical axis acceleration data were used to determine SPAcc, as most acceleration was found along that axis and in line with the longitudinal axis of the smartphone; no rotation correction was performed to align the smartphone’s local coordinate system with the global coordinate system as only temporal data was of interest [23,24]. SPAcc data were sample interpolated to 100 Hz using the Matlab “interp1” function, as the sampling rate was not constant. The gravity bias was removed from the accelerometery data, and the data were multiplied by −1 to correct for the upside-down orientation of the smartphone. Two data streams were created: one stream was kept in raw form for nonlinear measure calculations, and a second stream was filtered and used for linear measure calculations. Filtering was done using a fourth-order, low-pass Butterworth filter; a 16 Hz cutoff frequency was selected using residual analysis [34,35].

#### 2.3.1. Right-Heel Contact Locations

**Figure 1:**
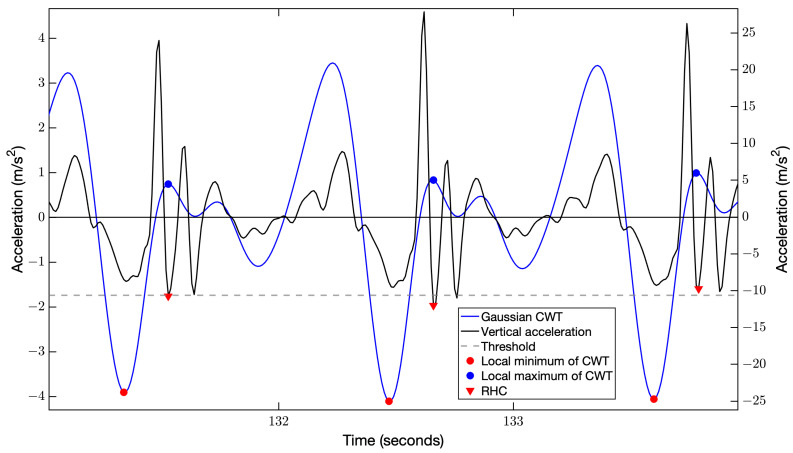
Illustration of the method used to determine right-heel contact (RHC) events within the filtered smartphone accelerometer data. The vertical acceleration (black line) is first integrated and then differentiated using a Gaussian continuous wavelet transform (CWT) (blue line). The local minimums are located (red circles) within the CWT signal and these indices are used to locate the local maximums (blue circles) within the CWT signal by searching forward relative to each local maximum. The horizontal dashed line represents the threshold of local minimum crossings for the CWT signal. Finally, the RHC events (red triangles) are located within the vertical acceleration signal as the local minimum relative to each blue circle.

The local minimum following the first local maximum in the vertical acceleration profile was considered the right-heel contact event [23,24,29] (Figure 1). A processing protocol similar to previous research was used to initially approximate right-heel contact events within the filtered vertical acceleration profile using a Gaussian continuous wavelet transform (CWT) [36]. The CWT provides simultaneous time and frequency analysis by decomposing the signal to localized oscillations of a defined wavelet shape and scale and was used as a smoothing and differentiating function [37].

The following steps were used to locate right-heel contact events within the SPAcc:

1. Remove low-frequency drift from the previously low-pass filtered vertical acceleration using a high-pass filter with a 0.1 Hz cutoff frequency [25].
2. Smooth the signal by integrating the low- and high-pass filtered vertical acceleration signal using the Matlab function “Cumtrapz”, creating a vertical velocity signal.
3. Differentiate the vertical velocity signal using a Gaussian CWT with scale = 12, selected visually due to the close proximity of the peaks in the CWT signal to that of the local minimums in the vertical acceleration signal (Figure 1).
4. Locate the local minimums within the CWT signal based on the rules that the time between each local minimum is separated by greater than 0.8 s and below a threshold value set to 40% of the median of 10 minimum CWT values. The separation value of 0.8 s was considered the lower limit of a healthy adult’s stride time [38], while the threshold value was selected following visual inspection of several trials.
5. Locate the local maximums relative to the local minimums within the CWT signal.
6. Locate the local minimums (right-heel contact events) within the vertical acceleration signal relative to each local maximum within the CWT signal (Figure 1).

After the identification of right-heel contact locations within the filtered vertical acceleration signal (steps 1–6, above), these location indices were then used to locate right- heel contact events within the raw vertical acceleration signal. Specifically, a three-frame offset from the indices of the filtered right-heel contact locations was first created and then a search forward relative to those offsets within the raw vertical acceleration signal was performed to locate the local minimums. The three-frame offset was selected to account for any shift in the signal that may have occurred due to filtering. All right-heel contact events located in the SPAcc were visually inspected to ensure accuracy.

#### 2.3.2. Walking Bout Segmentation

**Figure 2:**
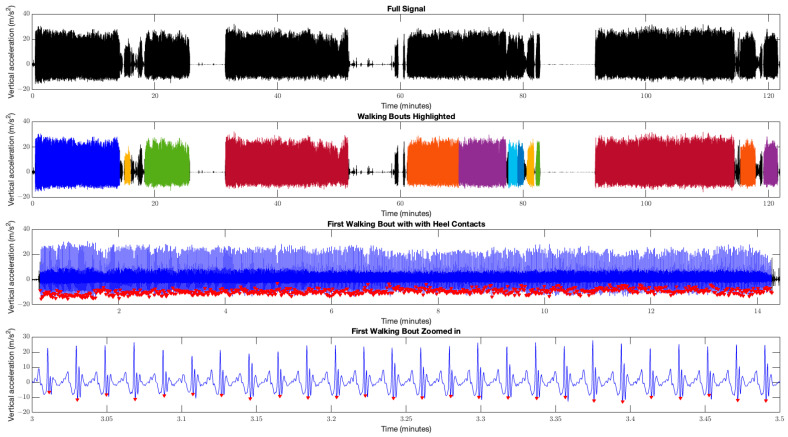
Representative plots of smartphone accelerometer data recorded on a young adult during free-living walking. Top represents the vertical acceleration signal recorded during the entire collection protocol. Top middle represents segmentation of the different walking bouts throughout the collection protocol with each colour (except black) denoting a separate walking bout identified and containing a minimum of 20 consecutive strides. Note that between 60 and 80 min, the orange and purple bouts as well as the light blue and blue bouts were successfully delineated despite the pause in walking behaviour being only approximately 3.5 s. Bottom middle represents the first walking bout, with right-heel contact events denoted by red triangles. Bottom represents a magnified section of the first walking bout.

Walking bouts were identified using the criterion that bouts are separated by periods of time greater than 1.8 s between consecutive right-heel contacts. Only walking bouts greater than 24 consecutive right-heel contact events (i.e., 24 strides) were selected. To ensure a steady-state gait was achieved, the first and last two strides of each bout were removed. Therefore, walking bouts of 20 or more strides were considered for analysis, as they were considered the recommended minimum number to reliably estimate stride-time variability [39] (Figure 2). The ISI series for each bout was calculated as the time difference between consecutive right-heel contact events, and then used for dependent measures calculations.

### 2.4. Dependent Measures

The following measures were calculated on all identified walking bouts: mean stride time (xISI) (s), stride time SD (s), and stride-time coefficient of variation (COV; %). The following measures were calculated on walking bouts of more than 255 strides: FSI, ApEn, and SaEn. The MSE (scales 1 to 4) and CI (SaEn versus scale area under the curve), SPD (strides), and EnHL (strides) were calculated on walking bouts of 800 strides based on the greatest number of strides completed within a single bout by all participants (Table 2).

Calculation of DFA: The ISI series was first integrated and then divided into non-overlapping boxes, or segments, of equal length (n). A least-squares line of best fit was applied within each box and then the integrated stride-time series was detrended by subtracting the line of best fit within each box. The root mean square (RMS) was then calculated for each box length and the average RMS was calculated across all boxes. This process was repeated with a range of box-size lengths (n = 10–40). A logarithmic transformation was applied to the plot of box length (n) versus RMS to create a log–log plot. Lastly, the slope of the line of best fit in the log–log plot provided the FSI value [40].

Calculation of ApEn and SaEn: The ApEn algorithm applied a sliding window to the stride-time series to determine the probability that short sequences of data points of vector length m were repeated within similarity criterion level r. The SaEn algorithm was similar to ApEn, except that the self-matching bias was removed. The input parameters, m and r, were selected as 2 and 0.15 multiplied by the SD of the stride-time series, respectively, based on the qualitative analysis of plotting different combinations of m and r parameters used for entropy calculation (Figure S1 in Supplementary Materials) [41,42].

Calculation of MSE: The coarse-graining process was applied to the stride-time series of increasing scale value as described in Costa et al. [16]. Scale values 1 to 4 were used and specified the number of data points averaged to obtain each value of the newly generated stride-time series. Afterwards, the SaEn was calculated using the stride-time series created at each scale and the SaEn (*y*-axis) was plotted against the scale (*x*-axis).

Calculation of CI: The area under the SaEn versus the scale curve (scales 1 to 4) was calculated using the Matlab function “trapz”. Only up to scale 4 was used to calculate CI to ensure 200 strides (800 strides/scale 4) were contained in the largest scale [41].

Calculation of SPD: SPD identifies the stride count at which the FSI value diverges from statistical persistence toward a value not different from uncorrelated noise. The original stride-time series was first reshaped 100 times using a custom-built Matlab function so that the ordering between two subsequent stride-time values was positionally increased with each reshaping to create 100 new ISI series, gradually increasingly randomizing the original series with each new ISI series. For example, reshaping 2 would result in an ISI series with the order of stride-time values consisting of every second value, relative to the original series. This can be described visually as follows: original ISI series: [1.13 s, 1.15 s, 1.17 s, 1.20 s, 1.22 s], reshaped 2: [1.13 s, 1.17 s, 1.22 s, 1.15 s, 1.20 s]. The FSI, using the DFA, was calculated for each reshaped time series yielding 100 FSI values. One hundred randomized time series were then created by a random permutation of the data points in the original time series using the Matlab function “randperm”. The FSI for each randomized time series ${(FSI}_{Rand})$ was calculated and the mean ${FSI}_{Rand}$ and corresponding SD ${FSI}_{Rand}$ were used to construct a critical limit, as follows:

(1) $$Critical\;limit=mean{FSI}_{Rand}+(2\times SD{FSI}_{Rand})$$

Lastly, the FSI of each reshaped time series (*y*-axis) was plotted against a stride order separation number (*x*-axis; 1–100) and the stride number at which the FSI value was less than the critical limit was selected as the SPD value. The reshaping method and SPD algorithm have been published in detail elsewhere [17].

Calculation of EnHL: EnHL estimates the number of strides taken before predictability in the time series is reduced by half. The SaEn from the original ISI series (${SaEn}_{original}$), as well as the reshaped SaEn ${(SaEn}_{reshape})$ and randomized mean SaEn (${SaEn}_{rand}$) as described above for SPD, were calculated. Each ${SaEn}_{reshape}$ was then normalized using the following equation:

(2) $$Normalized\;SaEn=\frac{{SaEn}_{reshape}-{SaEn}_{original}}{{SaEn}_{rand}-{SaEn}_{original}}$$

Finally, the normalized SaEn values (*y*-axis) were plotted against the stride order separation number (*x*-axis; 1–100) and the stride number at which the normalized SaEn value was less than 0.5 was selected as EnHL [43]. The EnHL algorithm has been published in detail elsewhere [17].

### 2.5. Statistical Analysis

Statistical analyses were performed using JMP v. 9.0 software (The SAS Institute, Cary, NC, USA). A frequency table was created to describe the number of walking bouts, the total number of strides, and the stride counts for the longest and shortest walking bouts for each participant (Table 2). One-way repeated-measures mixed-effects analysis of variance (ANOVA) was used to test the effect of group [YA/OA] on each dependent measure. Normality of data was assessed using distribution plots and Shapiro–Wilk tests for each measure. Non-normal data were log-transformed prior to statistical analyses. Statistical significance level was set at *p* < 0.05.

## 3. Results

### 3.1. Frequency Table

Description of the number and length of walking bouts identified using the SPAcc for each participant. Of note is the range of each measure across participants in both groups, which reflects the “free walking” context of this study, consistent with the instructions of “to behave as they normally would during a typical mall visit” provided to each participant at the beginning of each experiment.

**Table 2:** The number of walking bouts and stride-count characteristics for each participant captured with the smartphone accelerometer system during the two-hour collection protocol. Note: a walking bout contains a minimum of 20 consecutive strides after removing the first two and last two strides.

Participant	Walking Bouts	Total Strides	Longest Bout (Strides)	Shortest Bout (Strides)
**Older Adults**				
1	14	4091	823	21
2	20	4117	997	25
3	14	5286	1522	26
4	20	4439	1255	21
5	7	4515	1159	29
6	10	3933	963	23
7	5	1711	800	23
8	12	5774	2148	48
9	26	4593	816	22
10	12	5083	2218	25
11	10	3559	999	25
**Young Adults**				
1	12	4416	831	24
2	13	4520	1163	27
3	17	5455	1666	24
4	8	4879	1211	39
5	5	3927	1231	301
6	14	4045	948	24
7	9	5626	3322	34
8	11	4298	1831	21
9	8	3077	921	36
10	5	5204	3047	383
11	6	3527	928	21
12	9	5684	2186	21
13	10	3991	1017	24

### 3.2. Linear Measures

A significant group difference was found for stride time SD (F(1,22) = 5.03, *p* < 0.04) and COV (F(1,22) = 5.51, *p* < 0.03). Stride time SD and COV were significantly lower for YA compared with OA. No significant group difference was found for xISI (F(1,22) = 0.02, *p* = 0.89). All descriptive statistics are presented in Table 3.

### 3.3. Nonlinear Measures

A significant group difference was found for SaEn (F(1,22) = 6.5, *p* < 0.02), scale 1 (F(1,22) = 4.66, *p* = 0.04), scale 3 (F(1,22) = 7.38, *p* < 0.02), and SPD (F(1,22) = 5.04, *p* < 0.04). SaEn and scale 1 were significantly lower for YA compared with OA, while scale 3 and SPD were significantly greater for YA compared with OA. No significant group differences were found for FSI (F(1,22) = 0.58, *p* = 0.45), ApEn (F(1,22) = 0.08, *p* = 0.78), scale 2 (F(1,22) = 0.08, *p* = 0.78), scale 4 (F(1,22) = 1.02, *p* = 0.32), CI (F(1,22) = 1.37, *p* = 0.25), and EnHL (F(1,22) = 3.32, *p* = 0.08). All descriptive statistics are presented in Table 3.

## 4. Discussion

The current study explored the differences in linear and nonlinear gait measures between healthy young and older adults using a smartphone accelerometer system during overground walking in a free-living environment. Our results partially confirmed the hypotheses, showing significant age-related differences in several gait measures. These findings suggest that smartphone accelerometers could be a viable option for estimating gait patterns in ecologically valid settings while placed in the user’s pant pocket. Importantly, this study is the first to our knowledge that has demonstrated such findings, highlighting the potential for an inexpensive and user-friendly telehealth instrument to monitor gait dynamics remotely over extended periods of time.

### 4.1. Linear Measures

The OA group exhibited greater stride-time variability compared with the YA, consistent with previous research in controlled environments [44], suggesting reduced stability. However, both age groups revealed greater variability than reported in earlier studies involving treadmill or controlled overground walking [44,45]. This increase in variability could be linked to the unconstrained nature of the current study’s walking protocol, which most likely involved frequent speed adjustments to navigate environmental obstacles [46,47,48,49]. This variability may not necessarily reflect decreased gait stability but instead the more dynamic walking condition used in the current study. Therefore, interpretation of gait variability must consider the context in which the data were collected [19,48].

Tasks that impose greater cognitive and attentional demands such as dual-task walking and obstacle negotiation increase gait variability compared with single-task walking [50,51]. For example, dual-task walking has been found to increase stride-time COV [52,53] as well as stride-to-stride variability in gait velocity [54], suggesting fewer attentional resources allocated toward controlling gait, as attentional resources are provided to the performance of the secondary cognitive task. Although not explicitly tested, it is reasonable to assume that similar demands (i.e., increased decision-making) existed in the free-living walking scenario used in the current study. The findings suggest that free-living walking may provide a more accurate representation of linear variability, potentially requiring updates to normative data for stride-time variability. Furthermore, stride-time variability may be considered an important measure for detecting differences between healthy age groups within the free-living environment.

### 4.2. Nonlinear Measures

Both age groups exhibited greater mean FSI values compared with previous studies, especially for the OA group [1,44]. The lack of significant difference in statistical persistence between groups was unexpected and may be attributed to the fact that most OA participants were physically active [55]. Alternatively, the free-living environment could have elicited an upregulation in adaptive behaviour in response to increased environmental constraints and task demands, shifting FSI values closer to the middle of the OMV continuum approaching optimal fractality (FSI = 1.0) [11,13]. For example, previous research has reported FSI values of 0.7 and 0.9 during symmetric and asymmetric treadmill walking, respectively, consistent with an adaptive response to the perturbed walking condition [11]. The adaptability in the stepping pattern could reflect an increase in functional relevance of gait strategies, as the walker was sufficiently challenged to optimize the long-term correlations found in the current study to navigate the real world, compared with a controlled walking protocol providing consistent stimuli and redundant feedback. Therefore, the free-living environment may have prescribed an increase in statistical persistence, similar to the matching of heel-contact timing to the visual or auditory fluctuating timing imperative during treadmill walking [56,57]. To elucidate the contribution of the free-living environment, an assessment of baseline FSI values could be performed prior to real-world walking. Moreover, the FSI values found in the current study may reflect an improved representation of the locomotor system’s statistical persistence, although more work must be done to corroborate these findings.

The mean SaEn at scale 1 was found to be greater among OA, compared with YA, suggesting greater stride-time irregularity and trending toward the disordered side of the OMV continuum. These findings are consistent with the notion that less-regular stride patterns, such as those found in people with Parkinson’s disease, may indicate decreased gait adaptability [58]. Although no between-group difference was found at scale 2, both groups increased SaEn, after which YA demonstrated greater SaEn at scale 3 compared with OA. The finding of greater SaEn at greater scales suggests greater complexity and putatively the generation of a large repertoire of beneficial stepping strategies, in line with previous work reporting that older adult non-fallers demonstrated greater MSE-based measures compared with fallers during free-living walking [59]. These findings highlight the importance of multiscale analysis and the relationship between greater gait complexity suggesting greater gait adaptability in the free-living environment. The CI did not differ between age groups, which aligns with previous research that reported no significant difference between fallers and non-fallers for trunk or shank accelerations during overground walking [60]. This finding suggests that CI may not be sensitive enough to distinguish between high-functioning groups, as the faller group was considered low-risk. Although only scales 1 to 4 were investigated in the current study, future research should collect longer walking trials to assess greater scales using the SPAcc.

The mean SPD was found to be greater for YA compared with OA, which suggests that OA experience an earlier breakdown in stride-time complexity compared with YA, aligning with the age-related Loss of Complexity hypothesis. The SPD values ranged from 23 to 31 strides, exceeding previous treadmill-walking research among YA (~19 strides) when estimated on a similar number of strides [18]. Interestingly, that study also reported a mean FSI value of 1.07, which is greater than that of the current study (0.93–0.95). These findings suggest treadmill walking may induce a more structured stride-time fractality, increasing statistical persistence beyond optimal, while free-living walking induces a slightly less structured stride pattern, delaying the decay of statistical persistence, despite a lower FSI value. Previous work has reported a quadratic relationship between step- length symmetry and FSI during asymmetric treadmill walking, with greater step-length symmetry performed when FSI values were between 0.9 and 1.0 [11]. Therefore, limits to the structure of variability may exist, such that too little or too much structure may compromise gait performance, while a sufficiently challenged walker may require greater adaptability expressed as a bounded limit of statistical persistence and explain the lower FSI yet greater SPD values in the current study.

A trend between age groups showed that stride-time predictability was halved at 6 and 10 strides for YA and OA, respectively, suggesting OA may maintain stride-time regularity over a greater number of strides. Moreover, mean EnHL values in the current study were found to be slightly lower (~12 strides) compared with previous treadmill walking research [18], which may indicate that free-living walking is a more challenging task. These findings suggest that SPD may be more sensitive to age-related changes in gait patterns and the breakdown in stride-to-stride fluctuations compared with EnHL. Future research should explore EnHL across different coarse-grained scales to better understand the relationship between SPD and EnHL measures. A limitation of the current study was the lack of direct environmental context and walking intensity during each walking bout identified, increasing the difficulty of interpreting the associated variability. Future research should implement video recording equipment and other biofeedback tools (i.e., heart rate monitors) to provide additional information about the walking environment and intensity.

## 5. Conclusions

This study is the first to reveal age-related differences in both linear and nonlinear gait variability measures derived from a SPAcc in an ecologically valid setting. The findings show significant age-related differences in several gait parameters, yet these differences may not only be due to aging but reflect the nature of free-living walking. The absence of age-related differences in gait complexity (FSI and CI) may indicate that free-living walking provides an improved representation of gait variability across the populations investigated. Moreover, the free-living protocol may have shifted the structure of variability to a more adaptable stride pattern for both age groups, compared with previous laboratory-based estimates, to accommodate the increased challenge associated with walking ‘in the wild’. However, more work is required to determine the contribution of the free-living environment to gait dynamics. These results imply that the SPAcc is a viable option for remote monitoring of gait dynamics when carried in the user’s pant pocket. Future research should leverage the SPAcc to investigate gait patterns across various adult populations and over extended periods of time to monitor gait adaptability and potentially assess fall risk remotely.

## Figures and Tables

**Table 1:** Participant characteristics.

Participant	FoF	Fall History	ABC Score (%)	Physically Active	Type of Physical Activity
**Older Adults**					
1	No	0	99	No	N/A
2	No	0	95	Yes	Walking
3	No	0	99	Yes	Walking
4	No	0	92	Yes	Walking/hockey
5	No	0	98	Yes	Walking
6	No	0	93	Yes	Walking
7	Somewhat	0	74	Yes	Walking
8	No	1	78	Yes	Aerobics
9	No	1	92	Yes	Walking/cycling/aqua fitness
10	No	1	95	Yes	Walking/cycling
11	No	0	94	Yes	Walking
**Young Adults**					
1	No	0	99	Yes	Resistance training/walking
2	No	0	99	Yes	Resistance training
3	No	0	100	Yes	Resistance training/soccer
4	No	0	100	Yes	Dance
5	No	0	99	Yes	Resistance training/soccer
6	No	0	99	Yes	Resistance training/soccer/volleyball
7	No	0	100	Yes	Walking
8	No	0	98	Yes	Resistance training/running/hockey
9	No	0	98	Yes	Walking
10	No	0	99	Yes	Resistance training/soccer
11	No	0	96	Yes	Walking
12	No	0	99	No	N/A
13	No	0	100	Yes	Walking

Notes: FoF = fear of falling; fall history = number of falls in the previous 12 months; ABC = Activities-specific Balance Confidence Scale; physically active = 150 min or more of weekly moderate-to-vigorous physical activity.

**Table 3:** Mean ± SD and ANOVA result of each gait measure calculated using the stride-time series estimated from the smartphone accelerometer system for each group.

Measure	Bout Length (Strides)	Older Adults	Young Adults	Difference	*F*	*p*-Value
xISI (s)	>19	1.124 ± 0.083	1.128 ± 0.078	−0.004	0.02	0.89
Stride-time SD (s)	>19	0.047 ± 0.021	0.039 ± 0.019	0.008	5.03	<0.04 *
Stride-time COV (%)	>19	4.16 ± 1.76	3.47 ± 1.58	0.69	5.51	<0.03 *
FSI	>255	0.93 ± 0.14	0.95 ± 0.10	−0.02	0.58	0.45
SaEn	>255	1.89 ± 0.27	1.70 ± 0.15	0.19	6.5	<0.02 *
ApEn	>255	1.35 ± 0.18	1.37 ± 0.15	−0.01	0.08	0.78
SaEn Scale 1	800	1.86 ± 0.26	1.70 ± 0.15	0.16	4.66	0.04 *
SaEn Scale 2	400	2.10 ± 0.37	2.14 ± 0.23	−0.04	0.08	0.78
SaEn Scale 3	266	1.80 ± 0.35	2.12 ± 0.48	−0.32	7.38	<0.02 *
SaEn Scale 4	200	1.80 ± 0.16	1.90 ± 0.28	−0.10	1.02	0.32
CI Scale 4	800	5.77 ± 0.82	6.05 ± 0.73	−0.28	1.37	0.25
SPD (strides)	800	23 ± 11	31 ± 7	−8	5.04	<0.04 *
EnHL (strides)	800	10 ± 11	6 ± 5	4	3.32	0.08

DF(1,22). xISI = mean stride time; SD = standard deviation; COV = coefficient of variation; FSI = fractal scaling index; SaEn = sample entropy; ApEn = approximate entropy; CI = complexity index; SPD = statistical persistence decay; EnHL = entropic half-life. Statistical significance *p* < 0.05 *.

## Data Availability

The data presented in the current study will be made available upon request from the corresponding author.

## Supplementary Materials

The following supporting information can be downloaded at https://www.mdpi.com/article/10.3390/s24227175/s1: Figure S1: Qualitative analysis for the determination of vector comparison length, m, and similarity criteria, r, for approximate and sample entropy calculations.

## Abbreviations

ApEn	Approximate entropy
CI	Complexity index
EnHL	Entropic half-life
FSI	Fractal scaling index
ISI	Inter-stride interval
MSE	Multiscale entropy
OMV	Optimal movement variability
SaEn	Sample entropy
SPAcc	Smartphone accelerometer
SPD	Statistical persistence decay
