# Supplementary material for: Monitoring Age-Related Changes in Gait Complexity in the Wild with a Smartphone Accelerometer System
Source: Sensors (Basel). 2024 Nov 8;24(22):7175. doi: 10.3390/s24227175 (PMC11598579; doi:10.3390/s24227175)
Supplement: Supplementary file 1 [file sensors-24-07175-s001.zip › sensors-3220318-supplementary.pdf]

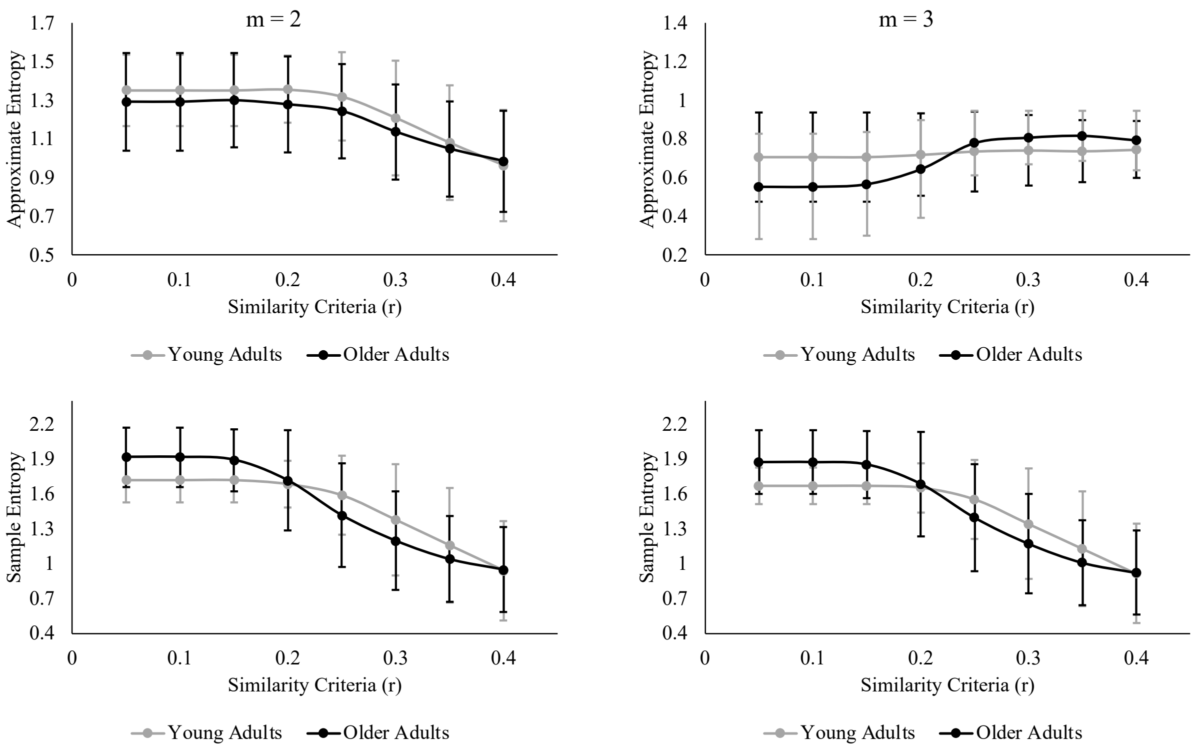

Figure S1. Qualitative analysis for the determination of vector comparison length, m, and similarity criteria, r, for approximate and sample entropy calculations. The approximate and sample entropy values (y-axes) were calculated as the average across all trials for each age group and are plotted against the different r values (x-axes) that were tested. As demonstrated by the line plots, the entropy values are fairly flat and with parameter consistency between groups across r values with m = 2 between 0.05 and 0.15 for approximate entropy and sample entropy; both groups trended downward as r increased between 0.25 to 0.4. The young adult group was considered the control group. Following this analysis, m = 2 and r = 0.15 were selected for both sample and approximate entropy calculations. Note, for all entropy calculations, each r value tested was multiplied by the standard deviation of the inter-stride interval series of each walking bout greater than 255 consecutive strides. Error bars represent standard deviation.
